# Chronic Antioxidant Capacity Loss in Anterior Chamber Environment After Iridectomy

**DOI:** 10.1167/tvst.12.5.4

**Published:** 2023-05-01

**Authors:** Shogo Arimura, Kentaro Iwasaki, Takuma Neo, Yusuke Orii, Takehiro Matsumura, Yoshihiro Takamura, Masaya Oki, Masaru Inatani

**Affiliations:** 1 Department of Ophthalmology, Faculty of Medical Sciences, University of Fukui, Yoshida, Fukui, Japan; 2 Department of Applied Chemistry and Biotechnology, Graduate School of Engineering, University of Fukui, Fukui, Japan; 3 Life Science innovation center, University of Fukui, Fukui, Japan

**Keywords:** trabeculectomy, iridectomy, aqueous humor, ascorbic acid, antioxidant capacity

## Abstract

**Purpose:**

To compare the ascorbic acid concentration and total antioxidant capacity in the aqueous humor of pigmented Rex rabbits after sham operation (control), iridectomy, and trabeculectomy.

**Methods:**

Pigmented Rex rabbits were divided into control, iridectomy, and trabeculectomy groups and followed up for 12 months after surgery. Ascorbic acid concentration and total antioxidant capacity in the aqueous humor, intraocular pressure, and the occurrence of cataracts were examined in each group.

**Results:**

The ascorbic acid concentration and total antioxidant capacity after iridectomy and trabeculectomy were significantly lower at one week and at one, six, and 12 months after operation than those in the control group (*P* ≤ 0.03). Ascorbic acid concentration was positively and significantly correlated with total antioxidant capacity in the aqueous humor (*P* < 0.01). Compared to the control and the iridectomy groups, intraocular pressure in the trabeculectomy group was significantly lower at one week and at one and six months after surgery (one week: *P* < 0.01 and *P* < 0.01, respectively; one month: *P* < 0.01 and *P* = 0.03, respectively; six months: *P* = 0.03). Histological findings in the iridectomy and trabeculectomy groups included the appearance of vacuoles in the lens at six and 12 months after surgery.

**Conclusions:**

Iridectomy causes a sustained decrease in ascorbic acid concentration, followed by a long-term decrease in the total antioxidant capacity within the aqueous humor.

**Translational Relevance:**

The animal model possibly predicts the vulnerability focusing on the antioxidant level in the anterior chamber environment after trabeculectomy and iridectomy per se in clinical settings.

## Introduction

Glaucoma and cataracts are the leading causes of blindness worldwide^1^^,^^2^ and often coexist because both are age related. Treatment of cataracts is surgical, whereas treatment for glaucoma can be performed by medical, laser, surgical, or combination therapy. Trabeculectomy is a typical filtration surgery for glaucoma that is effective in lowering intraocular pressure (IOP).^3^^–^^5^ However, one of the disadvantages of trabeculectomy is postoperative cataract progression.^6^^–^^8^ The cause of cataract progression after trabeculectomy is unclear. It has been postulated that cataract progression might be caused by postoperative corticosteroid use,^9^ hypotony, and the collapse of the anterior chamber,^10^^,^^11^ or it might be the result of changes in the dynamics of the aqueous humor.^12^

An alternative filtration surgery, Ex-PRESS filtration surgery, uses a unique device designed to stabilize the filtered aqueous volume to prevent postoperative complications derived from overfiltration. Previously, we compared postoperative complications after Ex-PRESS filtration surgery with those after trabeculectomy during a two-year follow-up.^13^ Interestingly, cataract progression was significantly slower in the Ex-PRESS filtration surgery group than in the trabeculectomy group. Ex-PRESS filtration surgery does not require iridectomy during the surgical procedure. We hypothesized that iridectomy might change the content of aqueous humor nourishing the lens, resulting in cataract progression.

Factors that disrupt homeostasis in the anterior chamber such as inflammation,^14^ lipid abnormalities,^15^ glycation,^16^ and oxidative stress^17^^–^^19^ are closely linked to cataract progression. Oxidative stress, including free radicals and reactive oxygen species (ROS), is known to cause cellular damage^20^ and protein degeneration.^21^ Ascorbic acid in the aqueous humor is present at much higher concentrations than in plasma^22^^,^^23^ to prevent cataract progression^24^^,^^25^ as an antioxidant. Total antioxidant capacity (TAC) has been measured as an indicator of oxidative resistance of antioxidants in the human body.^26^ Ascorbic acid is expected to account for a high proportion of TAC in aqueous humor.^27^ Therefore we aimed to determine whether iridectomy per se would alter the antioxidant environment in the aqueous humor by measuring ascorbic acid concentration and total antioxidant capacity.

## Methods

Pigmented male Rex rabbits weighing 2.0 to 2.5 kg (Japan SLC Co. Ltd, Shizuoka, Japan) were acclimated to their surroundings for at least one week. All rabbits were kept under pathogen-free conditions at 23°C ± 1°C, 60% ± 10% humidity, and 12 hours of light with 12 hours of darkness. Animals were housed with free access to water and food (RC4; Oriental Yeast Co. Ltd., Tokyo, Japan) throughout the day. The maintenance and experimentation of animals conformed to the guidelines of the Association for Research in Vision and Ophthalmology Statement for the Use of Animals in Ophthalmic and Vision Research during all procedures. A total of 96 right eyes of 96 rabbits were divided 1:1:1 into the sham operation (control), trabeculectomy, and iridectomy groups. Each group included 32 eyes. The postoperative measurement points were at one week and at one, six, and 12 months. Eight eyes were assigned to each postoperative measurement point in each group for sample collection. General anesthesia was administered by intramuscular injection of a mixture of ketamine (35 mg/kg) and xylazine (5 mg/kg) during surgery in the surgery room of the Department of Biological Resources at the University of Fukui.

Aqueous humor samples were collected to analyze whether there were significant differences in ascorbic acid concentration and TAC among the groups at one week and at one, six, and 12 months after surgery. In addition, the lenses of rabbit were enucleated for histological examination among the groups at one week and at one, six, and 12 months after surgery. The presence of cataracts was visually assessed using a portable slit lamp before enucleation. The enucleated lenses were cut to a thickness of 3 µm using a microtome for pathological specimen preparation with paraffin embedding. The specimens were observed under a microscope (IX70; Olympus, Tokyo, Japan). Conventional hematoxylin and eosin staining was used to examine the postoperative lens pathology. IOP was measured one day before surgery and at one week and at one, six, and 12 months after surgery using TONOVET (Icare, Helsinki, Finland) in conscious rabbits.

### Surgical Techniques

Conjunctival sacs were disinfected prior to all the surgeries with 10% polyvinyl alcohol iodine (Nitten Pharmaceutical, Nagoya, Japan) diluted ×6.

#### Trabeculectomy

A 7-mm conjunctival incision was made along the corneal limbus. A square scleral flap with a side length of 3 mm was created along the corneal limbus of the upper sclera. Three sponges soaked in mitomycin C (0.4 mg/mL; Kyowa Kirin, Tokyo, Japan) were applied under the conjunctival and scleral flaps for four minutes. The subconjunctival tissue and scleral flap were then washed with 10 mL normal saline solution (Otsuka, Tokyo, Japan). The trabecular meshwork below the scleral flap was excised. The iris was pulled from the excisional wound, and peripheral iridectomy was performed. The scleral flap was sutured using 10-0 nylon, maintaining aqueous humor filtration. The conjunctival wound was tightly closed using 10-0 nylon.

#### Iridectomy

A 7-mm conjunctival incision was made along the corneal limbus. A slit knife was inserted into the sclera near the corneal limbus, and the iris was removed using forceps. The incision was sutured using 10-0 nylon.

#### Sham Operation

A 7-mm conjunctival incision was made along the corneal limbus. A slit knife was inserted into the sclera near the corneal limbus, and the iris was touched with forceps without cutting the iris.

Eye drops of 1.5% levofloxacin hydrate as an antibacterial drug were administered three times a day after the surgeries in the sham control, the iridectomy, and the trabeculectomy groups for a week. Corticosteroid eye drops were not used after the surgeries because corticosteroid administration affects cataract progression and postoperative inflammation.

### Samples

Before sample collection, rabbits were anesthetized with an intramuscular injection of a mixture of ketamine (35 mg/kg) and xylazine (5 mg/kg). The aqueous humor was collected with a 30 G needle connected to an insulin syringe and stored in a −80°C freezer until measurement. After sample collection, the rabbits were euthanized with an intravenous injection of 10 mL sodium pentobarbital. The lens was enucleated immediately after euthanasia, then soaked in 4% PFA, and stored in a −4°C freezer until tissue staining. A total of eight eyes were collected at each measurement point in each group for the purpose of measuring ascorbic acid concentration and TAC in the aqueous humor, and examining histological changes of the lens.

### Ascorbic Acid Measurement

The samples were diluted 50 times and then measured using an assay kit (Ascorbic Acid Quantification Kit; Bio Vision Inc., Milpitas, CA, USA). The concentration of ascorbic acid was determined by fluorometric methods (Ex/Em = 535/587 nm). The assay could detect the amount of 0.01 to 10 nmol of ascorbic acid per assay.

### Total Antioxidant Capacity Measurement

TAC in the aqueous humor was measured using a commercially available kit (Total Antioxidant Status Assay Kit; MEGA TIP San. Tic. Ltd, Mücahitler, Turkey). Antioxidants in the kit reduce dark blue-green ABTS radicals to a colorless reduced ABTS form. The change in the absorbance at 660 nm was related to the total antioxidant level of the sample. The assay is calibrated with a stable antioxidant standard solution, traditionally called Trolox equivalent, which is a vitamin E analog.

### Statistical Analysis

In each group, normality was measured using the Shapiro-Wilk test. To compare means among the three groups, an analysis of variance (ANOVA) test was first performed, followed by a post-hoc test using the Tukey-Kramer method. The proportional correlation between ascorbic acid concentration and TAC in aqueous humor was presented as a scatter diagram analyzed by Spearman's correlation analysis. SPSS version 27.0 (IBM Corp., Armonk, NY, USA) was used for statistical analysis. For all tests, a *P* value less than 0.05 was considered significant. Preoperative data are shown as the mean ± standard deviation. Postoperative data are shown as the mean ± standard error.

### Additional Experiment

We performed an additional experiment to clarify the mechanism of antioxidant capacity loss in the aqueous humor postoperatively. Eight right eyes of eight rabbits were divided 1:1 into the sham-surgery group and anterior lamellar excision of peripheral iris group, which was the left posterior stroma group (defined as “anterior iridectomy” group). Each group included four eyes. We measured and compared the concentrations of ascorbic acid and various cytokines including vascular endothelial growth factor-A, tumor necrosis factor-α (TNF-α), monocyte chemoattractant protein-1 (MCP-1), and interleukin-6 (IL-6) in aqueous humor between the sham-surgery group and the anterior iridectomy group with assay and enzyme-linked immunosorbent assay kits. Mann-Whitney U test was performed to analyze the significance between the groups. The postoperative measurement points were at one week and at one and two months. The aqueous humor was collected repeatedly in the same eye at each measurement point. The surgical techniques for the sham-surgery group, anesthetic methods for sample collection, and sample storage were the same as described in this “Methods” section, in the “Surgical techniques” and “Samples” subsections. For the surgical technique in anterior iridectomy group, a 7-mm conjunctival incision was made along the corneal limbus. A slit knife was inserted into the sclera near the corneal limbus, and the anterior lamellar of peripheral iris was excised using forceps, which left the posterior stroma. The incision was sutured with 10-0 nylon.

## Results

### Ascorbic Acid Concentration

The comparison of the means of the ascorbic acid concentrations among the three groups showed significant differences with ANOVA at all measurement points (*P* < 0.01). The post-hoc test showed that the ascorbic acid concentrations after iridectomy and trabeculectomy were significantly lower at all measurement points at one week and at one, six, and 12 months after surgery than in the control group (one week: *P* < 0.01 and *P* < 0.01, respectively; one month: *P* < 0.01 and *P* = 0.03, respectively; six months: *P* < 0.01 and *P* < 0.01, respectively; 12 months: *P* < 0.01 and *P* < 0.01, respectively) (Table 1).

**Table 1.** Comparison of Ascorbic Acid Concentration (mM) Among the Groups

				*P* Value		
Group	CONT	IRI	TRAB	CONT vs. IRI	CONT vs. TRAB	IRI vs. TRAB
1 W	0.89 ± 0.03	0.46 ± 0.03	0.50 ± 0.03	<0.01^*^	<0.01^*^	0.61
1 M	0.82 ± 0.07	0.42 ± 0.06	0.53 ± 0.08	<0.01^*^	<0.03^*^	0.52
6 M	0.84 ± 0.03	0.45 ± 0.05	0.35 ± 0.06	<0.01^*^	<0.01^*^	0.32
12 M	0.92 ± 0.05	0.49 ± 0.03	0.56 ± 0.04	<0.01^*^	<0.01^*^	0.46

CONT, control; IRI, iridectomy; TRAB, trabeculectomy; W, week; M, month.

A Tukey-Kramer analysis was used to determine *p* values.

* Statistical significance (*P* < 0.05).

### Total Antioxidant Capacity

**Figure 1.**
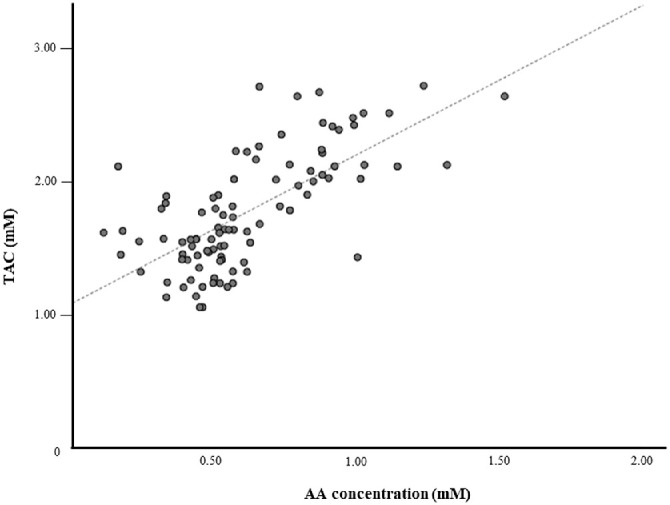
Correlation of ascorbic acid concentration and total antioxidant capacity. Spearman's correlation analysis revealed significantly positive correlation between AA concentration and TAC in all samples (n = 96, *r* = 0.66, *P* < 0.01). AA, ascorbic acid.

In the comparison of TAC among the three groups, there were significant differences with ANOVA at all measurement points (*P* < 0.01). The post-hoc test showed that the TAC after iridectomy and trabeculectomy was significantly lower at all measurement points at one week and at one, six, and 12 months after surgery compared to the control group (one week: *P* < 0.01 and *P* < 0.01, respectively; one month: *P* < 0.01 and *P* < 0.01, respectively; six months: *P* < 0.01 and *P* < 0.01, respectively; 12 months: *P* < 0.01 and *P* < 0.01, respectively) (Table 2). Spearman's correlation analysis revealed a significant positive correlation between the ascorbic acid concentration and TAC in all samples (n = 96, *r* = 0.66, *P* < 0.01) (Fig. 1).

**Table 2.** Comparison of TAC (mM) Among the Groups

				*P* Value		
Group	CONT	IRI	TRAB	CONT vs. IRI	CONT vs. TRAB	IRI vs. TRAB
1 W	2.14 ± 0.08	1.44 ± 0.08	1.47 ± 0.09	<0.01^*^	<0.01^*^	0.97
1 M	2.18 ± 0.05	1.53 ± 0.11	1.53 ± 0.10	<0.01^*^	<0.01^*^	1.00
6 M	2.27 ± 0.10	1.38 ± 0.06	1.60 ± 0.11	<0.01^*^	<0.01^*^	0.25
12 M	2.50 ± 0.07	1.51 ± 0.03	1.68 ± 0.05	<0.01^*^	<0.01^*^	0.06

CONT, control; IRI, iridectomy; TRAB, trabeculectomy; W, week; M, month.

A Tukey-Kramer analysis was used to determine *P* values.

* Statistical significance (*P* < 0.05).

### Postoperative IOPs

Table 3 shows the IOPs in the control, iridectomy, and trabeculectomy groups. In the comparison of IOPs among the three groups, there were significant differences with ANOVA at all measurement points (*P* < 0.01). The post-hoc test showed that IOPs after trabeculectomy were significantly lower at one week and at one and six months after surgery compared to the control group and the iridectomy group (one week: *P* < 0.01 and *P* < 0.01, respectively; one month: *P* < 0.01 and *P* = 0.03, respectively; six months: *P* = 0.03).

**Table 3.** The Comparison of IOPs (mm Hg) Among the Groups

				*P* Value		
Group	CONT	IRI	TRAB	CONT vs. IRI	CONT vs. TRAB	IRI vs. TRAB
Pre-op	13.9 ± 1.6	12.4 ± 1.1	13.1 ± 1.6	0.12	0.56	0.56
1 W	14.1 ± 0.6	13.7 ± 0.4	8.6 ± 0.3	0.60	<0.01^*^	<0.01^*^
1 M	13.9 ± 0.6	13.1 ± 0.7	10.9 ± 0.3	0.63	<0.01^*^	0.03^*^
6 M	14.4 ± 0.8	14.1 ± 0.6	12.0 ± 1.0	0.95	0.03^*^	0.61
12 M	14.0 ± 0.5	13.6 ± 0.7	13.8 ± 0.6	0.90	0.96	0.99

CONT, control; IRI, iridectomy; TRAB, trabeculectomy; Pre-op, preoperative; W, week; M, month.

Tukey-Kramer analysis was used to determine *P* values.

* Statistical significance (*P* < 0.05).

### Cataract Progression

**Figure 2.**
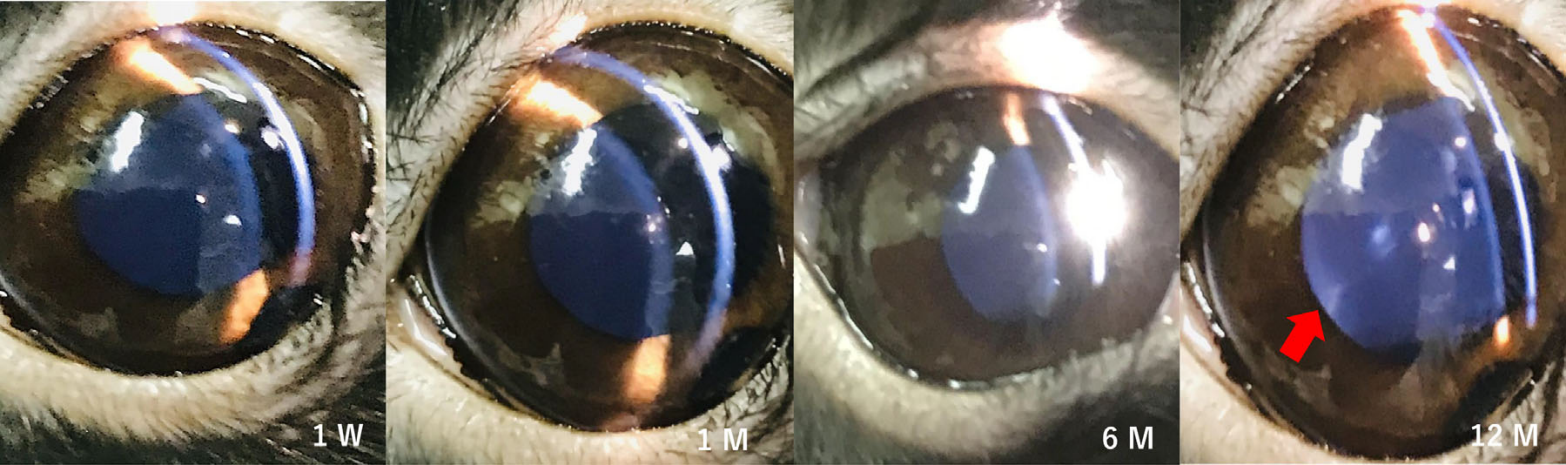
Anterior chamber examination by a slit lamp in the iridectomy group. Cortical opacity was observed 12 months postoperatively. The *red arrow* indicates the area of cortical opacity in the lens.

**Figure 3.**
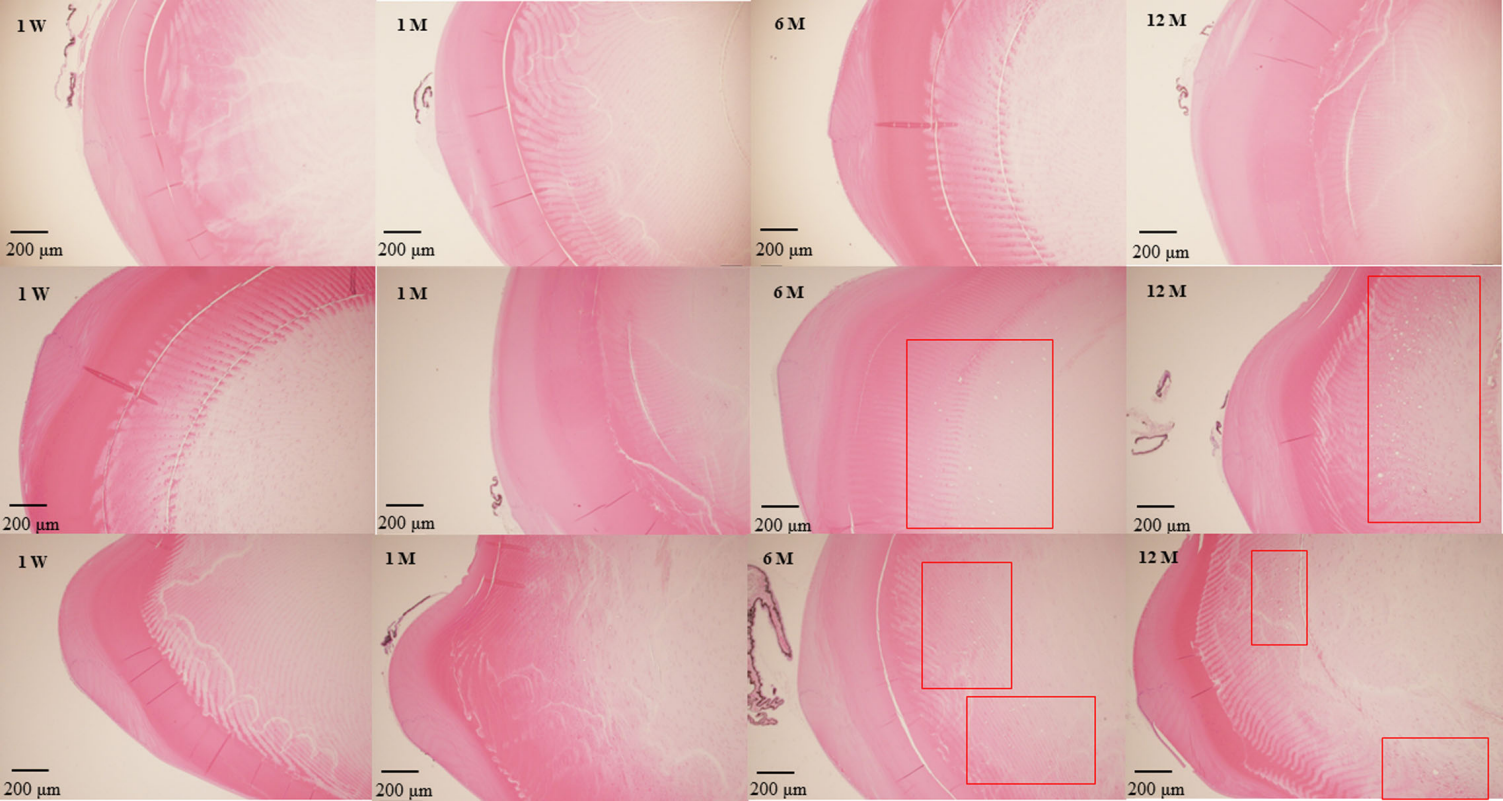
Histological changes in the lens after the surgeries. The *top row* represents the control group. The *middle row* shows the results of the iridectomy group. The *bottom row* shows the results of the trabeculectomy group. There were vacuole changes between the cortex and nucleus in the lens at six and 12 months in the iridectomy and trabeculectomy groups after surgery. Vacuole changes are indicated in *red*.

Postoperative anterior chamber photographs of the iridectomy group were obtained using a portable slit lamp (Fig. 2). Cortical opacity in the lens was visually observed at 12 months after surgery. The results of hematoxylin and eosin staining in the control group, the iridectomy group, and the trabeculectomy group are shown Figure 3. There were no cataractous changes in the lens either at one week or one month in each group, but numerous vacuole changes between the cortex and nucleus were observed at six and 12 months in the iridectomy and the trabeculectomy group.

### Additional Experiment

The ascorbic acid and IL-6 concentrations of anterior iridectomy group were significantly lower at one week and at one and two months after surgery than those of the sham-surgery group (one week: *P* = 0.03; one month: *P* = 0.03; two months: *P* = 0.03). The MCP-1 concentration in anterior iridectomy group was significantly lower at one week and at two months after surgery than those in the sham-surgery group (one week: *P* = 0.03; two months: *P* = 0.03). There were no significant differences in vascular endothelial growth factor-A and TNF-α concentrations between the groups. The numerical data are presented in Supplementary Tables S1 to S5.

## Discussion

The present study showed that the concentration of ascorbic acid, a reductant agent in aqueous humor, and TAC were significantly lower in the iridectomy and the trabeculectomy group than in the control group. Additionally, regression analysis showed that the ascorbic acid concentration had a significant positive correlation with the TAC (*P* < 0.01). The antioxidant environment associated with ascorbic acid and TAC in the aqueous humor is altered after iridectomy and trabeculectomy. There have been reports that focused on oxidative stress and glaucoma, including serum oxidative stress level,^28^^,^^29^ damage to retinal neurons,^30^ retinal ganglion cell death,^31^ glaucoma severity,^32^ and supplements for glaucoma,^33^ although no studies have examined oxidative stress levels after trabeculectomy. This study provides new insights related to the vulnerability in anterior chamber environment after iridectomy, focusing on antioxidant condition in aqueous humor.

Ascorbic acid is a powerful reducing agent of ROS in the anterior chamber environment. ROS are generated as a result of oxygen consumption by air respiration,^34^ exposure to radiation,^35^ ultraviolet light,^36^ smoking,^37^ inflammatory reaction,^38^ and surgery.^39^ ROS include sequential one-electron reduction products of O_2_, such as O_2_−, H_2_O_2_, and OH. The OH group of ascorbic acid donates H+ to convert OH into H_2_O. The immediate postoperative decline in ascorbic acid concentration and TAC in the iridectomy and the trabeculectomy groups may be due to the consumption of ascorbic acid to remove OH from postoperative transient inflammation. Ascorbic acid concentration and TAC in the iridectomy and the trabeculectomy groups remained significantly lower than those in the control group until 12 months after surgery. The long-term loss of ascorbic acid and TAC may be related to chronic inflammation due to iris damage. Aketa et al.^40^ demonstrated that iris damage elevated proinflammatory cytokines such as IL-α, IL-6, IL-8, TNF-α, MCP-1, and interferon-γ in the collection of aqueous humor samples six months to 10 years after intraocular surgeries,^41^ including laser peripheral iridectomy and trabeculectomy. Our additional experiment showed that the anterior lamellar excision of the peripheral iris sustained an increase of inflammatory cytokine levels including MCP-1 and IL-6 and a decrease of ascorbic acid concentration in the aqueous humor for two months after surgery. Furthermore, previous studies have shown that the interaction between iris pigment epithelial cells and the components of the aqueous humor maintains the homeostasis of the immune system in the anterior chamber, because the iris pigment epithelium has immunosuppressive and immunomodulatory effects.^42^^–^^45^ Inflammatory responses inevitably occur when cells and tissues are exposed to stress or injury, as when infection occurs, because some receptors on immune cells detect damage-associated molecular pattern molecules released from damaged cells and tissues.^46^^,^^47^ Proteomic approaches^48^ from human samples suggest that iris atrophy results in blood aqueous barrier disruption and elevated protein levels, with concurrent complement activation and oxidative stress in the aqueous humor. This study suggests that failure of immunomodulatory factors caused by iris damage disturbs the microenvironment in the aqueous humor, resulting in long-term elevation in the levels of aqueous inflammatory cytokines accompanied by ascorbic acid consumption for ROS removal.

Another reason for the long-term loss of ascorbic acid and TAC may be related to alterations in the aqueous humor flow after iridectomy. Razzak et al.^49^ hypothesize that cataracts are caused by trauma to the lens due to the continuous flow of aqueous humor through a surgical fistula created by trabeculectomy. It is possible that chronic trauma to the lens because of the alteration of aqueous flow may have led to the sustained consumption of ascorbic acid and TAC because ascorbic acid is absorbed by the lens and protects it from oxidation.^50^ However, in our additional experiment, there was a significant decrease of ascorbic acid concentration with a concurrent increase of inflammatory cytokines in the aqueous humor after surgery without direct aqueous humor flow from the posterior to anterior chamber for two months after surgery. Therefore we consider that the long-term antioxidant capacity loss is due to the prolonged inflammation rather than changes in the aqueous humor flow.

In the current study, cataracts were associated with iridectomy and trabeculectomy in slit-lamp examination and pathological specimens. To the best of our knowledge, no study has focused on postoperative oxidative stress and cataract progression after iridectomy and trabeculectomy. Despite the relationship between oxidative stress levels and cataract progression,^17^^–^^19^ studies to date have been limited to diet,^51^ epidemiology,^52^ and plasma oxidative stress levels.^53^ Few reports have mentioned changes in the antioxidant capacity of aqueous humor, with which the lens is in direct contact. Tsao et al.^27^ reported that ascorbic acid and TAC levels in the aqueous humor have a significant negative correlation with cataract severity, which is consistent with our results.

In addition, vacuoles were histologically observed between the cortex and nucleus of the lens at six and 12 months after surgery in the present study, whereas observation using the portable slit showed cortical cataractous changes at 12 months only. The areas of lens opacity after glaucoma surgery are controversial, with some studies reporting nuclear opacity,^13^ others reporting posterior subcapsular cataract,^54^ and still others stating that all types of cataracts progress.^6^ Our data indicate that cataract development after trabeculectomy begins with small vacuolar changes between the cortex and nucleus. Whether this change is specific to the long-term loss of antioxidant capacity requires further study.

In this study, trabeculectomy significantly reduced IOPs compared to the control and iridectomy groups at one week and at one and six months after surgery. In contrast, IOPs were not significantly different between the iridectomy and control groups. Although a shallow anterior chamber and low IOP have been reported as risk factors for cataract progression after trabeculectomy,^10^^,^^11^ the iridectomy group did not encounter hypotony in the present study. This suggests that postoperative antioxidant levels in the aqueous humor with concurrent cataract progression are independent of IOP and anterior chamber collapse. Our data indicate that peripheral iris defects cause the consumption of antioxidants in the aqueous humor.

This study has several limitations. Other antioxidants, such as glutathione and uric acid, were not measured because of the limited amount of aqueous humor samples available. The sample size for the additional experiments was small, and other inflammatory and anti-inflammatory cytokines need to be investigated. A longer measurement period is necessary to confirm the long-term loss of ascorbic acid and the elevation levels of the cytokines in the aqueous humor after surgery. In addition, the TAC was not measured in the additional experiment because of the small volume of the aqueous humor that can be collected from one eye. We will have to conduct comprehensive studies to evaluate the effect of postoperative inflammatory cytokines on antioxidant levels in the aqueous humor by multivariate analysis. Although this study visually examined the appearance of cataracts, there were no objective data regarding lens opacity. Some reports have shown the progression of cataracts numerically using Image J with photographs taken under a microscope^55^ or with an anterior segment imaging device.^13^ The correlation between lens opacity and antioxidant levels in the aqueous humor should be analyzed in the future.

In conclusion, we observed a long-term loss in ascorbic acid concentration and TAC in the aqueous humor and cataract progression after iridectomy and trabeculectomy. This study provides a new information related to the vulnerability of anterior chamber environment after iridectomy, especially in antioxidant conditions.

## Supplementary Material

Supplement 1

## References

[1] Quigley HA, Broman AT. The number of people with glaucoma worldwide in 2010 and 2020. *Br J Ophthalmol*. 2006; 90: 262–267. 16488940 10.1136/bjo.2005.081224 PMC1856963

[2] Keenan TD, Salmon JF, Yeates D, et al. Trends in rates of trabeculectomy in England. *Eye* 2009; 23: 1141–1149. 18617913 10.1038/eye.2008.195

[3] Leske MC, Heijl A, Hyman L, Bengtsson B. Early Manifest Glaucoma Trial: Design and baseline data. *Ophthalmology*. 1999; 106: 2144–2153. 10571351 10.1016/s0161-6420(99)90497-9

[4] Musch DC, Gillespie BW, Lichter PR, et al. Visual field progression in the collaborative initial glaucoma treatment study: The impact of treatment and other baseline factors. *Ophthalmology*. 2009; 116: 200–207. 19019444 10.1016/j.ophtha.2008.08.051 PMC3316491

[5] Radcliffe NM, Musch DC, Niziol LM, et al. The effect of trabeculectomy on intraocular pressure of the untreated fellow eye in the collaborative initial glaucoma treatment study. *Ophthalmology*. 2010; 117: 2055–2060. 20570363 10.1016/j.ophtha.2010.02.016 PMC3334525

[6] AGIS investigators. The advanced glaucoma intervention study: 8 Risk of cataract formation after trabeculectomy. *Arch Ophthalmol*. 2001; 119: 1771–1779. 11735786 10.1001/archopht.119.12.1771

[7] Lazaro C, Benitez-del-Castillo JM, Castillo A, et al. Lens fluorophotometry after trabeculectomy in open angle glaucoma. *Ophthalmology*. 2002; 109: 76–79. 11772583 10.1016/s0161-6420(01)00865-x

[8] Adelman RA, Brauner SC, Afshari NA, Grosskreutz CL. Cataract formation after initial trabeculectomy in young patients. *Ophthalmology*. 2003; 110: 625–629. 10.1016/S0161-6420(02)01769-4 12623833

[9] Mohan R, Muralidharan AR. Steroid induced glaucoma and cataract. *Indian J Ophthalmol**.* 1989; 37: 13–16. 2807493

[10] Robin AL, Ramakrishnan R, Krishnadas R, et al. A long-term dose response study of mitomycin in glaucoma filtration surgery. *Arch Ophthalmol**.* 1997; 115: 969–974. 9258217 10.1001/archopht.1997.01100160139001

[11] Costa VP, Smith M, Spaeth GL, Gandham S, Markovitz B. Loss of visual acuity after trabeculectomy. *Ophthalmology**.* 1993; 100: 599–612. 8493002 10.1016/s0161-6420(93)31597-6

[12] Clarke MP, Vernon SA, Sheldrick JH. The development of cataract following trabeculectomy. *Eye (Lond)**.* 1990; 4: 577–583. 2226987 10.1038/eye.1990.80

[13] Arimura S, Miyake S, Iwasaki K, et al. Randomized Clinical Trial For Postoperative Complications After Ex-PRESS implantation versus trabeculectomy with 2-year follow-Up. *Sci Rep*. 2018; 8(1): 16168. 30385884 10.1038/s41598-018-34627-w PMC6212395

[14] Foster CS, Barrett F. Cataract development and cataract surgery in patients with juvenile rheumatoid arthritis-associated uveitis. *Ophthalmology**.* 1993; 100: 809–817. 8510892 10.1016/s0161-6420(93)31568-x

[15] Huang L, Grami V, Marrero Y, et al. Human lens phospholipid changes with age and cataract. *Invest Ophthalmol Vis Sci*. 2005; 46: 1682–1689. 15851569 10.1167/iovs.04-1155

[16] Stevens VJ, Rouzer CA, Monnier VM, Cerami A. Diabetic cataract formation: Potential role of glycosylation of lens crystallins. *Proc Natl Acad Sci USA*. 1978; 75: 2918–2922. 275862 10.1073/pnas.75.6.2918 PMC392677

[17] Spector A . Oxidative stress-induced cataract: Mechanism of action. *FASEB J*. 1995; 9: 1173–1182. 7672510

[18] Shui YB, Fu JJ, Garcia C, et al. Oxygen distribution in the rabbit eye and oxygen consumption by the lens. *Invest Ophthalmol Vis Sci**.* 2006; 47: 1571–1580. 16565394 10.1167/iovs.05-1475

[19] Vinson JA. Oxidative stress in cataracts. *Pathophysiology*. 2006; 13: 151–162. 16765571 10.1016/j.pathophys.2006.05.006

[20] Ryter SW, Kim HP, Hoetzel A, et al. Mechanisms of cell death in oxidative stress. *Antioxid Redox Signal*. 2007; 9: 49–89. 17115887 10.1089/ars.2007.9.49

[21] Cabiscol E, Tamarit J, Ros J. Oxidative stress in bacteria and protein damage by reactive oxygen species. *Int Microbiol*. 2000; 3: 3–8. 10963327

[22] Taylor A, Jacques PF, Nadler D, et al. Relationship in humans between ascorbic acid consumption and levels of total and reduced ascorbic acid in lens, aqueous humor, and plasma. *Curr Eye Res*. 1991; 10: 751–759. 1914507 10.3109/02713689109013869

[23] Senthilkumari S, Talwar B, Dharmalingam K, et al. Polymorphisms in sodium-dependent vitamin C transporter genes and plasma, aqueous humor and lens nucleus ascorbate concentrations in an ascorbate depleted setting. *Exp Eye Res**.* 2014; 124: 24–30. 24815519 10.1016/j.exer.2014.04.022

[24] Lim JC, Caballero Arredondo M, Braakhuis AJ, Donaldson PJ. Vitamin C and the Lens: New Insights into Delaying the Onset of Cataract. *Nutrients*. 2020; 12: 3142. 33066702 10.3390/nu12103142 PMC7602486

[25] Tessier F, Moreaux V, Birlouez-Aragon I, Junes P, Mondon H. Decrease in vitamin C concentration in human lenses during cataract progression. *Int J Vitam Nutr Res*. 1998; 68: 309–315. 9789763

[26] Fraga CG, Oteiza PI, Galleano M. In vitro measurements and interpretation of total antioxidant capacity. *Biochim Biophys Acta*. 2014; 1840: 931–934. 23830861 10.1016/j.bbagen.2013.06.030

[27] Tsao YT, Wu WC, Chen KJ, et al. An assessment of cataract severity based on antioxidant status and ascorbic acid levels in aqueous humor. *Antioxidants (Basel)*. 2022; 11: 397. 35204279 10.3390/antiox11020397 PMC8869206

[28] Yamada E, Himori N, Kunikata H, et al. The relationship between increased oxidative stress and visual field defect progression in glaucoma patients with sleep apnoea syndrome. *Acta Ophthalmol*. 2018; 96(4): e479–e484. 29498225 10.1111/aos.13693

[29] Tanito M, Kaidzu S, Takai Y, Ohira A. Correlation between systemic oxidative stress and intraocular pressure level. *PLoS One**.* 2015; 10(7): e0133582. 26186651 10.1371/journal.pone.0133582 PMC4506090

[30] Harada T, Harada C, Watanabe M, et al. Functions of the two glutamate transporters GLAST and GLT-1 in the retina. *Proc Natl Acad Sci USA*. 1998; 95: 4663–4666. 9539795 10.1073/pnas.95.8.4663 PMC22547

[31] Yang X, Hondur G, Tezel G. Antioxidant treatment limits neuroinflammation in experimental glaucoma. *Invest Ophthalmol Vis Sci*. 2016; 57: 2344–2354. 27127934 10.1167/iovs.16-19153 PMC4855827

[32] Yoshikawa T, Obayashi K, Miyata K, Saeki K, Ogata N. Association between the asymmetric dimethylarginine levels and glaucoma severity: A cross-sectional analysis of the LIGHT Study. *Invest Ophthalmol Vis Sci*. 2021; 62(4): 7. 10.1167/iovs.62.4.7 PMC8039475 33821880

[33] Himori N, Inoue Yanagimachi M, Omodaka K, et al. The effect of dietary antioxidant supplementation in patients with glaucoma. *Clin Ophthalmol*. 2021; 15: 2293–2300. 34113073 10.2147/OPTH.S314288 PMC8183457

[34] Murphy MP. How mitochondria produce reactive oxygen species. *Biochem J*. 2009; 417: 1–13. 19061483 10.1042/BJ20081386 PMC2605959

[35] Tominaga H, Kodama S, Matsuda N, Suzuki K, Watanabe M. Involvement of reactive oxygen species (ROS) in the induction of genetic instability by radiation. *J Radiat Res*. 2004; 45: 181–188. 15304958 10.1269/jrr.45.181

[36] de Jager TL, Cockrell AE, Du Plessis SS. Ultraviolet light induced generation of reactive oxygen species. *Adv Exp Med Biol*. 2017; 996: 15–23. 29124687 10.1007/978-3-319-56017-5_2

[37] Stampfli MR, Anderson GP. How cigarette smoke skews immune responses to promote infection, lung disease and cancer. *Nat Rev Immunol* 2009; 9: 377–384. 19330016 10.1038/nri2530

[38] Mittal M, Siddiqui MR, Tran K, Reddy SP, Malik AB. Reactive oxygen species in inflammation and tissue injury. *Antioxid Redox Signal*. 2014; 20: 1126–1167. 23991888 10.1089/ars.2012.5149 PMC3929010

[39] Yang Y, Bazhin AV, Werner J, Karakhanova S. Reactive oxygen species in the immune system. *Int Rev Immunol*. 2013; 32: 249–270. 23617726 10.3109/08830185.2012.755176

[40] Aketa N, Yamaguchi T, Suzuki T, et al. Iris damage is associated with elevated cytokine levels in aqueous humor. *Invest Ophthalmol Vis Sci*. 2017; 58: 42–51. 28475702 10.1167/iovs.17-21421

[41] Yamaguchi T, Higa K, Suzuki T, et al. Elevated cytokine levels in the aqueous humor of eyes with bullous keratopathy and low endothelial cell density. *Invest Ophthalmol Vis Sci*. 2016; 57: 5954–5962. 27820951 10.1167/iovs.16-20187

[42] Streilein JW, Bradley D. Analysis of immunosuppressive properties of iris and ciliary body cells and their secretory products. *Invest Ophthalmol Vis Sci*. 1991; 32: 2700–2710. 1894470

[43] Suzuma I, Mandai M, Suzuma K, et al. Contribution of E-selectin to cellular infiltration during endotoxin-induced uveitis. *Invest Ophthalmol Vis Sci*. 1998; 39: 1620–1630. 9699551

[44] Yoshida M, Kezuka T, Streilein JW. Participation of pigment epithelium of iris and ciliary body in ocular immune privilege. 2. Generation of TGF-β–producing regulatory T cells. *Invest Ophthalmol Vis Sci*. 2000; 41: 3862–3870. 11053287

[45] Lemaitre C, Thillaye-Goldenberg B, Naud MC, de Kozak Y. The effects of intraocular injection of interleukin-13 on endotoxin-induced uveitis in rats. *Invest Ophthalmol Vis Sci*. 2001; 42: 2022–2030. 11481267

[46] Zindel J, Kubes P. DAMPs, PAMPs, and LAMPs in immunity and sterile inflammation. *Annu Rev Pathol*. 2020; 15: 493–518. 31675482 10.1146/annurev-pathmechdis-012419-032847

[47] Murao A, Aziz M, Wang H, Brenner M, Wang P. Release mechanisms of major DAMPs. *Apoptosis*. 2021; 26(3-4): 152–162. 33713214 10.1007/s10495-021-01663-3 PMC8016797

[48] Yamaguchi T, Higa K, Yagi-Yaguchi Y. Pathological processes in aqueous humor due to iris atrophy predispose to early corneal graft failure in humans and mice. *Sci Adv**.* 2020; 6(20): eaaz5195. 32426498 10.1126/sciadv.aaz5195 PMC7220341

[49] Razzak A, al Samarrai A, Sunba MS. Incidence of posttrabeculectomy cataract among Arabs in Kuwait. *Ophthalmic Res*. 1991; 23: 21–23. 1870836 10.1159/000267081

[50] Kannan R, Stolz A, Ji Q, Prasad PD, Ganapathy V. Vitamin C transport in human lens epithelial cells: Evidence for the presence of SVCT2. *Exp Eye Res*. 2001; 73: 159–165. 11446766 10.1006/exer.2001.1024

[52] Chiu CJ, Taylor A. Nutritional antioxidants and age-related cataract and maculopathy. *Exp Eye Res*. 2007; 84: 229–245. 16879819 10.1016/j.exer.2006.05.015

[51] Mathew MC, Ervin AM, Tao J, et al. Antioxidant vitamin supplementation for preventing and slowing the progression of age-related cataract. *Cochrane Database Syst Rev*. 2012; 6(6): CD004567. 22696344 10.1002/14651858.CD004567.pub2 PMC4410744

[53] Cui YH, Jing CX, Pan HW. Association of blood antioxidants and vitamins with risk of age-related cataract: A meta-analysis of observational studies. *Am J Clin Nutr*. 2013; 98: 778–786. 23842458 10.3945/ajcn.112.053835

[54] Husain R, Aung T, Gazzard G, Foster PJ. Effect of trabeculectomy on lens opacities in an East Asian population. *Arch Ophthalmol*. 2006; 124: 787–792. 16769831 10.1001/archopht.124.6.787

[55] Kanada F, Takamura Y, Miyake S, et al. Histone acetyltransferase and Polo-like kinase 3 inhibitors prevent rat galactose-induced cataract. *Sci Rep*. 2019; 9(1): 20085. 31882756 10.1038/s41598-019-56414-x PMC6934598

