# Supplementary material for: Chronic Antioxidant Capacity Loss in Anterior Chamber Environment After Iridectomy
Source: Transl Vis Sci Technol. 2023 May 1;12(5):4. doi: 10.1167/tvst.12.5.4 (PMC10153582; doi:10.1167/tvst.12.5.4)
Supplement: Supplement 1 [file tvst-12-5-4_s001.pdf]

Supplementary Table S1. Comparison of ascorbic acid concentration (mM) between sham-surgery group and anterior iridectomy group.

	Sham-surgery	Anterior iridectomy	*p* value
1 week	0.86 (0.80 to 0.94)	0.53 (0.39 to 0.61)	0.03*
1 month	0.95 (0.72 to 1.08)	0.27 (0.20 to 0.40)	0.03*
2 months	0.86 (0.79 to 0.94)	0.24 (0.13 to 0.39)	0.03*

Data are shown as median (interquartile range from the first to the third quartile). Mann-Whitney U test. *, statistically significant ($p < 0.05$).

Anterior iridectomy group, anterior lamellar excision of peripheral iris group.

Supplementary Table S2. Comparison of VEGF-A (pg/ml) concentration between sham-surgery group and anterior iridectomy group.

	Sham-surgery	Anterior iridectomy	*p* value
1 week	50.5 (47.5 to 55.5)	51.5 (43.0 to 57.0)	0.89
1 month	56.5 (47.5 to 81.0)	63.5 (54.5 to 77.5)	0.69
2 months	54.0 (48.0 to 70.0)	89.5 (65.0 to 94.5)	0.34

Data are shown as median (interquartile range from the first to the third quartile). Mann-Whitney U test. *, statistically significant ($p < 0.05$).

VEGF-A, vascular endothelial growth factor-A; Anterior iridectomy group, anterior lamellar excision of peripheral iris group.

Supplementary Table S3. Comparison of TNF-α concentration (pg/ml) between sham-surgery group and anterior iridectomy group.

	Sham-surgery	Anterior iridectomy	*p* value
1 week	3.22 (2.67 to 4.50)	3.98 (2.91 to 4.60)	0.69
1 month	3.68 (3.25 to 4.34)	4.74 (4.09 to 5.38)	0.11
2 months	3.98 (3.22 to 4.61)	4.45 (3.64 to 4.98)	0.69

Data are shown as median (interquartile range from the first to the third quartile). Mann-Whitney U test. *, statistically significant ($p < 0.05$).

TNF-α, tumor necrosis factor-α; Anterior iridectomy group, anterior lamellar excision of peripheral iris group.

Supplementary Table S4. Comparison of MCP-1 concentration (pg/ml) between sham-surgery group and anterior iridectomy group.

	Sham-surgery	Anterior iridectomy	*p* value
1 week	37.1 (27.5 to 53.2)	171.5 (80.2 to 273.1)	0.03*
1 month	50.6 (42.8 to 62.6)	80.4 (47.1 to 116.0)	0.49
2 months	37.1 (27.5 to 41.6)	146.7 (71.6 to 241.3)	0.03*

Data are shown as median (interquartile range from the first to the third quartile). Mann-Whitney U test. *, statistically significant (p < 0.05).

MCP-1, monocyte chemoattractant protein-1; Anterior iridectomy group, anterior lamellar excision of peripheral iris group.

Supplementary Table S5. Comparison of IL-6 concentration (pg/ml) between sham-surgery group and anterior iridectomy group.

	Sham-surgery	Anterior iridectomy	*p* value
1 week	68.5 (59.5 to 77.9)	158.6 (114.7 to 199.7)	0.03*
1 month	62.3 (56.8 to 68.1)	187.3 (126.9 to 262.0)	0.03*
2 months	62.0 (56.3 to 74.8)	180.8 (155.2 to 222.3)	0.03*

Data are shown as median (interquartile range from the first to the third quartile). Mann-Whitney U test. *, statistically significant ($p < 0.05$).

IL-6, interleukin-6; Anterior iridectomy group, anterior lamellar excision of peripheral iris group.
